# Don’t Rock the Boat: How Antiphase Crew Coordination Affects Rowing

**DOI:** 10.1371/journal.pone.0054996

**Published:** 2013-01-30

**Authors:** Anouk J. de Brouwer, Harjo J. de Poel, Mathijs J. Hofmijster

**Affiliations:** 1 MOVE Research Institute Amsterdam, Faculty of Human Movement Sciences, VU University Amsterdam, Amsterdam, The Netherlands; 2 Center for Human Movement Sciences, University Medical Center Groningen, University of Groningen, Groningen, The Netherlands; McMaster University, Canada

## Abstract

It is generally accepted that crew rowing requires perfect synchronization between the movements of the rowers. However, a long-standing and somewhat counterintuitive idea is that out-of-phase crew rowing might have benefits over in-phase (i.e., synchronous) rowing. In synchronous rowing, 5 to 6% of the power produced by the rower(s) is lost to velocity fluctuations of the shell within each rowing cycle. Theoretically, a possible way for crews to increase average boat velocity is to reduce these fluctuations by rowing in antiphase coordination, a strategy in which rowers perfectly alternate their movements. On the other hand, the framework of coordination dynamics explicates that antiphase coordination is less stable than in-phase coordination, which may impede performance gains. Therefore, we compared antiphase to in-phase crew rowing performance in an ergometer experiment. Nine pairs of rowers performed a two-minute maximum effort in-phase and antiphase trial at 36 strokes min^−1^ on two coupled free-floating ergometers that allowed for power losses to velocity fluctuations. Rower and ergometer kinetics and kinematics were measured during the trials. All nine pairs easily acquired antiphase rowing during the warm-up, while one pair’s coordination briefly switched to in-phase during the maximum effort trial. Although antiphase interpersonal coordination was indeed less accurate and more variable, power production was not negatively affected. Importantly, in antiphase rowing the decreased power loss to velocity fluctuations resulted in more useful power being transferred to the ergometer flywheels. These results imply that antiphase rowing may indeed improve performance, even without any experience with antiphase technique. Furthermore, it demonstrates that although perfectly synchronous coordination may be the most stable, it is not necessarily equated with the most efficient or optimal performance.

## Introduction

Crew rowing is often used as an expedient example when referring to cooperation between multiple agents in a group and synchronization phenomena in general. Indeed, in rowing practice it is generally accepted that perfect synchronization between the rower’s movements is paramount for optimal performance of the crew as a whole [1], [2]. Curiously, a myth that has been roaming around for quite some time already, states that when rowers move *out*-of-phase with respect to each other this may actually be beneficial over the conventional in-phase (i.e., synchronous) pattern [3]–[5]. Indeed, crew rowing in out-of-phase coordination theoretically minimizes the power loss to velocity fluctuations of the shell within the rowing cycle, which may enhance average boat velocity. Although there are some anecdotic records that attempts to row out-of-phase were not successful, the reasons why such attempts failed are still based on speculation rather than experimental data. Therefore, this study experimentally probed the somewhat counterintuitive idea of out-of-phase rowing.

### Mechanical Power and Efficiency in Rowing

The goal in competitive rowing is to cover a 2000 m race distance in the shortest amount of time. Accordingly, to achieve maximum average boat velocity each rower or rowing crew aims to maximize power output and minimize power losses. In rowing, mechanical power is inevitably lost during the push-off with the blades, but also to velocity fluctuations of the shell within the rowing cycle. Shell velocity fluctuates for two reasons; (1) propulsion is not continuous and (2) the center of mass of the relatively heavy rower(s) moves relative to the boat over a considerable distance, causing the shell to accelerate in the opposite direction of the acceleration of the rower(s) [6], [7]. The mechanical power equation of rowing illustrates that these velocity fluctuations increase the total amount of power needed to overcome hydrodynamic drag, because this power is proportional to shell velocity cubed [7], [8]. In steady state rowing, where there is no net change in kinetic and potential energy of the rower(s) over any full rowing cycle, power needed to overcome hydrodynamic drag can be divided into useful power (related to average velocity: *P*_v_) and wasted power (lost to velocity fluctuations: *P*_Δv_). Total averaged power dissipated to drag (*P*_drag_) can thus be written as:
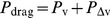
(1)


Unless indicated otherwise, all following mechanical power terms concern averages over one or more full rowing cycles in the steady state situation.

Velocity efficiency describes the fraction of power produced by the rower that is not lost to velocity fluctuations [8]. This efficiency is reported to be about 0.94 to 0.95, corresponding to a 5 to 6% power loss [7]–[9]. Minimizing velocity fluctuations while maintaining power output will result in an increase in velocity efficiency and, hence, higher average boat velocity. This offers an interesting possibility for performance enhancement as is widely recognized by scientists [3], [4], [6], [7], [10], [11]. As an example, the sliding-rigger (i.e., an on-board mechanical implement that minimizes shell velocity fluctuations) demonstrated to be highly successful in skiff rowing world championships from 1981 to 1983 [12]. However, this device was officially prohibited from rowing competition after 1983. Later it has been shown that altering conventional rowing technique can result in a small reduction of velocity fluctuations [6], [13]. Interestingly, for crew rowing, a much larger reduction in fluctuations can theoretically be achieved through a strategy in which pairs of rowers row out-of-phase [3], [4]. By this strategy, the net within-cycle movement of the crew’s mass with respect to the shell would become close to zero and the boat would be more continuously propelled. Interestingly, a mathematical analysis of *anti*phase rowing, using prescribed center of mass motion of the rowers relative to the boat and equal forces exerted on the blades, showed that the reduction of velocity fluctuations would result in a gain of about one boat length (3.0 s) for an eight rowing a 2000 m race [4].

Yet, several practical and theoretical issues have been raised that argued against out-of-phase rowing. For instance, Nolte [10] argued that it would require longer boats, adjustment of drive and recovery times and a complete change of ‘boat feel’ for the rowers, and the crew would have difficulty to mutually coordinate the alternating movements. The latter potential problem can be explained within the framework of coordination dynamics.

### Coordination Dynamics

Many (non-)living physical, biological and social systems show synchronization tendencies (e.g., [14]). In the 17^th^ century, Huygens [15] witnessed that two pendulum clocks on a wall that were initially uncoordinated, became coordinated over time with either in-phase (phase difference *φ*  = 0°) or antiphase (*φ*  = 180°) coordination, interacting through mechanical vibrations via the wall. Ample evidence from human cyclic interlimb coordination reveals the same two stable coordinative states, with antiphase being less stably performed than in-phase (e.g., [16], [17]). Most notably, coordination is less stable at higher movement frequencies, while at a particular critical frequency antiphase coordination becomes unstable and a sudden transition to the more stable in-phase pattern occurs. Other coordination modes than in-phase and antiphase are initially unstable and require considerable practice in order to become stable [18].

These coordination phenomena were accounted for by the well-known HKB model [19]. This model yields a potential function that captures the dynamics of the relative phase (*φ*) between two nonlinearly coupled limit-cycle oscillators [19]. The relative phase (*φ*) is formulated as
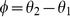
(2)with *θ*_2_ and *θ*_1_ depicting the phase angle of each oscillator, that is, where it resides in its cycle from 0° to 360°. The potential function reflects an attractor landscape with minima at *φ*  = 0° and *φ*  = 180°, defining the attractors for in-phase and antiphase, respectively. Importantly, it was demonstrated that the coordinative principles captured by the HKB model also apply to rhythmic coordination *between* two people (e.g., [20]–[22]). Thus, these coordination dynamics occur irrespective of whether the interaction between the components is mediated mechanically (clocks), neurally (interlimb), perceptually (interpersonal) or otherwise [23]. As a consequence of the lower stability of antiphase coordination, perturbations (e.g., external and/or related to required attentional costs [24], [25]) may lead to a transition from antiphase to in-phase coordination, especially at high movement rates [20]. Relating this to rowing, it is interesting to mention that in the late 1920s, newspapers were reporting of British rowing crews trying out-of-phase rowing by implementing a four-phase strategy (*φ  = *90°) in an eight (see [26]) and a three-phase strategy (*φ*  = 120°) with six rowers [5]. However, the attempts received a lot of criticism and they were never continued. This is not at all surprising, as 90° and 120° coordination patterns are inherently unstable [19] and, even after considerable practice, extremely difficult to maintain at high movement rates [18].

Knowing this, we considered the stable antiphase coordination (*φ*  = 180°) in crew rowing. For this strategy, the mechanics of rowing predict higher velocity efficiency and average boat velocity than for in-phase rowing. On the other hand, coordination dynamics predict that antiphase rowing is less stable than in-phase rowing, which possibly impedes these performance gains.

### Aim

The aim of this study was to test whether steady state crew rowing in antiphase coordination results in better performance compared to in-phase crew rowing. Given that higher efficiency does not necessarily imply better performance, we investigated how antiphase rowing affects total power production and useful power dissipation. To examine the power loss to velocity fluctuations that are caused by the forward-backward movements of the rowers, we used coupled ergometers that were put on slides to allow them to move back and forth (see *Experimental setup*). The kinematics of ergometer rowing are largely similar to those of on-water rowing [27], particularly for free-floating ergometers as used in the present study [28], [29]. Furthermore, we examined the effects of antiphase rowing on interpersonal coordinative performance by looking at the error and variability in relative phase angle.

## Materials and Methods

### Participants

Eighteen male rowers participated in the experiment (age 28±6 years, body height 1.90±0.07 m, body mass 83.5±9.5 kg, rowing experience at club level 5±3 years). Five pairs signed up for the experiment as a pair (i.e., being teammates), the other four pairs were composed based on availability for the experiment and matched for body mass. All participants provided written informed consent prior to participation.

### Ethics Statement

The experiment was approved by the ethics committee of the Faculty of Human Movement Sciences of VU University.

### Experimental Setup

**Figure 1:**
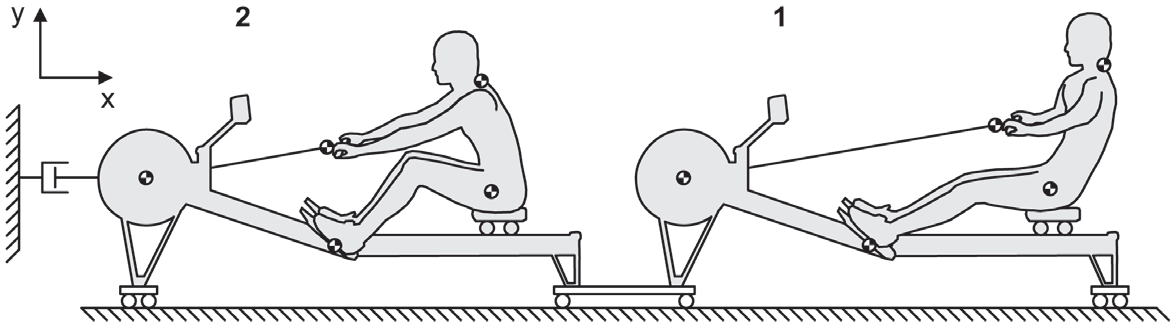
Schematic representation of the ergometer setup.



Trials were performed on two rowing ergometers serially placed on slides (Type D, Concept 2, USA) as schematically depicted in Figure 1. The resistance of the ergometer flywheels was set at an aerodynamic constant of 1.00 · 10^−4^ kg·m^2^ (i.e., drag factor 100). On standard rowing ergometers, there is no power loss to velocity fluctuations (*P*_Δv_) [13], thus, all the mechanical power that is produced by the rowers (*P*_rowers_) is transferred to the air-braked flywheels of the ergometers (*P*_flywheels_), simulating *P*_v_ of Equation 1. To introduce a power loss, movement of the ergometers was resisted by a servomotor that simulated *P*_Δv_ [13]. The servomotor was programmed such that *P*_Δv_ would be about 5–6%, despite of the smaller absolute velocity fluctuations due to the large mass of the ergometer setup (approximately 54 kg) compared to a double scull (27 kg). By providing a velocity-dependent resistive force (*F*_servo_), the servomotor acted as a linear damper approximating: *F*_servo_ = −50 · *v*_ergometers_, with *v*_ergometers_ the instantaneous velocity of the ergometer flywheels with respect to an earth-bound reference frame. Consequently, the equation for crew rowing on this ergometer setup can be written as

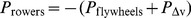
(3)with *P*_Δv_ the average power loss dissipated by the servomotor [13]. Equation 3 makes clear that performance is expressed by *P*_flywheels_ (i.e., useful power).

To determine the power terms of Equation 3, kinetics and kinematics of the rowers were recorded at 200 Hz during each trial. Handle forces of both ergometers were measured by two one-degree-of-freedom force transducers (AST, Germany), each placed between the chain and the handle. *F*_servo_ was measured by a one-degree-of-freedom force transducer (AM Cells, USA) mounted between the servomotor and the ergometers (Figure 1).

Kinematic variables were obtained using an Optotrak motion capture system (Northern Digital Inc., Canada) using two capturing units. Active infrared markers were placed on the flywheel, the handle force transducer and the foot stretcher of both ergometers, as well as on the hip (greater trochanter) and neck (at the level of vertebrae C5/C6) of each rower (Figure 1). Due to technical limitations of the Optotrak system the number of infrared markers was limited to maintain high sampling frequency. Therefore positions of the wrist, elbow, ankle and knee were estimated (see *Interpersonal coordination*). Segment lengths of the upper arm, lower arm, upper leg, lower leg, and trunk were measured to estimate segment and whole body center of mass (CoM) position.

### Protocol

To warm up and get familiar with the experimental setup, each pair started with rowing five minutes in in-phase coordination and five minutes in antiphase coordination at a self chosen stroke rate (about 20–24 min^−1^), including about 30 s of rowing at a high stroke rate (>30 min^−1^) for each condition. After a short break, each pair performed a two-minute in-phase and a two-minute antiphase trial in counterbalanced order with five minutes of rest in between. The pairs were instructed to row with maximal power output at a constant stroke rate of 36 min^−1^. Each rower received feedback about stroke rate and *P*_flywheel_ on a monitor (PM4, Concept 2, USA).

### Mechanical Power and Efficiency

Data were analyzed over an interval of 36 complete rowing cycles (approximately 60 s of rowing), starting from the first catch (i.e., the beginning of a rowing cycle) after 30 s of rowing. The catch was defined as the moment in time the handle started to move away from the flywheel. Data were filtered using a fourth order low-pass Butterworth filter with 15 Hz cut-off frequency. Data were analyzed in the sagittal plane using customized software (MATLAB, MathWorks, USA).

*P*_rowers_ was calculated according to Equation 3. First, instantaneous power dissipated by the ergometer flywheel of each rower was calculated according to
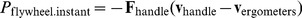
(4)with **F**_handle_ the handle force vector, and **v**_handle_ and **v**_ergometers_ the velocity vectors of handle and ergometers with respect to an earth-bound reference frame [13]. Instantaneous power dissipated by the servomotor was calculated as



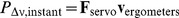
(5)Subsequently, the values of power dissipated by the flywheels and servomotor (*P*_flywheel1_, *P*_flywheel2_, and *P*_Δv_) were calculated by averaging instantaneous power over the 36 cycles. *P*_flywheels_ was calculated as *P*_flywheel1_+ *P*_flywheel2_. The values obtained for *P*_flywheels_ and *P*_Δv_ were substituted in Equation 3 to determine *P*_rowers_. Velocity efficiency (*e*_v_), the fraction of *P*_rowers_ that is not lost to velocity fluctuations, was calculated as
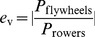
(6)(adapted from [13]). Finally, we calculated the total distance travelled by the ergometer setup per cycle (*s*_ergometers_), to provide insight in the effect of antiphase rowing on ‘boat movements’.

### Interpersonal Coordination

Forward-backward CoM movement of both rowers was estimated from the x- and y-positions of the CoMs of individual body segments according to Winter [30]. First, shoulder position was estimated on the line between hip and neck marker using pre-measured trunk length. Ankle position was estimated at a pre-measured distance from the foot stretcher, positioned on the line between foot stretcher marker and hip. The position of the wrist was estimated at a pre-measured distance from the handle marker on the line between handle marker and shoulder. The positions of the knee and elbow joint were reconstructed using the measured segment lengths and the distances between ankle and hip, and wrist and shoulder respectively. A pilot experiment verified that these estimations introduced only minor errors in the calculation of horizontal CoM position compared to the situation where ankle, knee, wrist and elbow positions were measured. The spatio-temporal relation between the rower CoM movements was expressed by the continuous relative phase (*φ*, see Equation 2), calculating the phase angles (*θ*_i_) of each individual CoM as
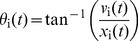
(7)with *x*_i_(*t*) the horizontal CoM position, *v*_i_(*t*) the horizontal CoM velocity normalized by dividing it by the angular frequency of each half cycle [31] and *i* depicting rower 1 or 2 (Figure 1). This normalization of CoM velocity was performed because in rowing the drive (cf. backward CoM movement) and recovery (cf. forward CoM movement) are typically not equal in duration [4], [11]. To provide an indication of this inharmonicity of CoM movement in both conditions, the mean ratio of the backward to forward CoM movement duration was calculated (*ratio*). This was based on instances of maximum and minimum excursions of the CoM trajectory.

For perfect in-phase and antiphase coordination, *φ* equals 0° and 180°, respectively. For antiphase rowing, shell velocity fluctuations would reduce to zero when the rowers’ movement trajectories mirror perfectly, that is, when deviation from 180° is zero. Therefore, the absolute deviation from 0 or 180° was calculated for each time sample and then averaged to obtain the absolute error of relative phase (AE*φ*), which expresses the accuracy with which the intended relative phase was achieved.

Furthermore, due to the nature of the rowing stroke, a rower spends more time around the finish than around the catch of the stroke [11]. Because this results in deviations from the intended relative phase of 0° or 180°, we also calculated a discrete measure of relative phase that is not sensitive to such inharmonicities. The relative phase based on point-estimates of peak CoM excursions near the catch of the stroke, was calculated for each full cycle as
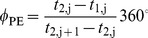
(8)where *t*_2,j_ and *t*_1,j_ indicate the time of the *j*th peak of the CoM of rower 2 and 1. The standard deviation of relative phase (SD*φ*_PE_) was calculated as a measure of coordination variability.

### Statistical Analysis

Paired samples t-tests were performed to investigate differences in mean stroke rate (SR), *P*_rowers_, *P*_flywheels_, *e*_v,_ AE*φ*, SD*φ*_PE_ and *ratio* between the in-phase and antiphase condition. A significance level of 0.05 was used.

## Results

All nine pairs easily managed rowing in antiphase coordination within the five-minute warm-up. One of the nine pairs showed a transition from antiphase to in-phase coordination during the maximum effort antiphase trial and was excluded from subsequent data analysis. Table 1 presents means and standard deviations of SR, *P*_rowers_, *P*_flywheels_, *e*_v_, AE*φ*, SD*φ*_PE_, and *ratio* over the 36 rowing cycles, and t-test statistics.

**Table 1:** Rowing performance in terms of mechanical power, velocity efficiency and interpersonal coordination (mean ± SD), and paired t-test statistics (*N*  = 8 pairs, *df*  = 7).

	In-phase	Antiphase	*t*	*p*
SR (min^−1^)	36.5±0.5	35.3±0.6	3.864	<.01
*P*_rowers_ (W)	731±73	740±80	0.723	0.493
*P*_flywheels_ (W)	−690±66	−734±81	3.349	<.05
*e*_v_	0.945±0.013	0.991±0.004	11.167	<0.001
*s*_ergometers_ (m)	0.96±0.11	0.33±0.06	12.180	<.001
AE*φ* (°)	7±1	24±5	11.001	<.001
SD*φ*_PE_ (°)	4±2	12±6	3.340	<.05
*Ratio*	1∶ 1.2±0.1	1∶ 1.2±0.1	1.528	0.170

### Mechanical Power and Efficiency

In the in-phase condition SR was slightly higher than the instructed 36 min^−1^, whereas in the antiphase condition SR was somewhat lower. The small but significant difference in SR between conditions did not result in a significant difference in *P*_rowers_ (Table 1). However, *P*_flywheels_ was significantly higher for antiphase rowing (mean difference 44 W, *P*_flywheels,antiphase_ >*P*_flywheels,in-phase_ for all pairs), which was mainly related to the significantly higher *e*_v_ in this condition. On average, *e*_v_ was 0.046 higher for antiphase rowing. Accordingly, *s*_ergometers_ was much smaller for antiphase rowing. These results show that during antiphase rowing a smaller fraction of *P*_rowers_ was lost to velocity fluctuations, consequently more power was transferred to the flywheels.

### Interpersonal Coordination

**Figure 2:**
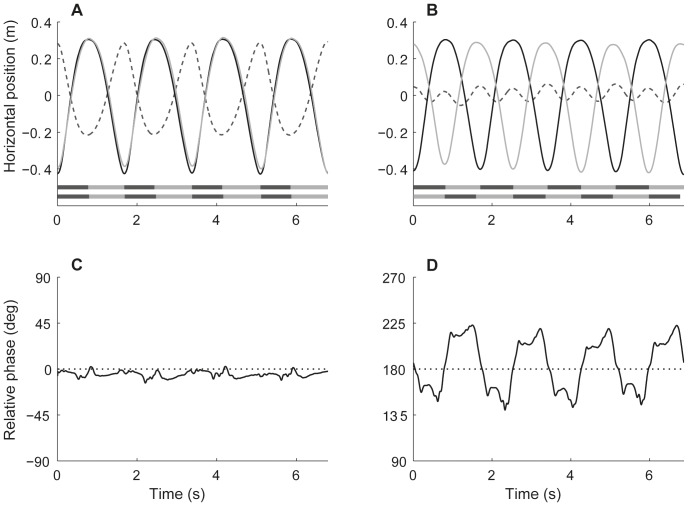
Interpersonal coordination. Top: an example of the center of mass (CoM) movement of two rowers (solid lines) and the movement of the ergometers (dashed line) during inphase (A) and antiphase rowing (B). The bars below the CoM movement indicate the duration of backward (cf. drive phase; dark grey) and forward (cf. recovery phase; light grey) CoM movement of both rowers. Bottom: continuous relative phase between the CoM movements during inphase (C) and antiphase rowing (D). The intended relative phase is displayed by the dotted lines.

Figure 2 shows an example of the movement of rowers’ CoMs and the ergometer during four strokes of in-phase (A) and antiphase rowing (B). As can be seen from the bars, the backward CoM movement was slightly faster than the forward CoM movement. However, this *ratio* did not differ significantly between in-phase and antiphase rowing (see Table 1). Further, note that the upper peaks in CoM movement are less sharp than the lower peaks, because the rowers spent more time around the finish than around the catch of the stroke. This is mainly because around the finish, only the arms of the rower move, while the relatively heavy trunk of the rower does not move. In Figure 2D, which shows an example of the continuous relative phase during antiphase rowing, it can be seen that this causes periodic fluctuations around the intended 180°. These fluctuations are less apparent in the continuous relative phase during in-phase rowing (Figure 2C).

In accordance, AE*φ* was significantly lower for in-phase rowing compared to antiphase rowing (Table 1), meaning that the deviations from the intended relative phase (0° and 180°) were smaller for in-phase rowing than for antiphase rowing. Similarly, SD*φ*_PE_ was significantly smaller for in-phase rowing, indicating lower coordinative variability compared to antiphase rowing.

## Discussion

This study examined whether steady state crew rowing in antiphase coordination results in better performance compared to in-phase crew rowing, by investigating total power production and useful power dissipation as well as interpersonal coordination. The first striking observation was that all nine pairs easily managed antiphase rowing on the coupled free-floating ergometers within a few strokes of the warm-up. Thus, also in rowing, two people can maintain stable in-phase as well as antiphase coordination with minimal practice (e.g., [20], [22]).

### Mechanical Power and Efficiency

In line with the expectations following from Equations 5 and 6, velocity efficiency was higher for antiphase crew rowing. The observed reduction in power loss compared to in-phase rowing was on average 4.6%, which is close to the hypothetical 5 to 6% gain that would occur when speed fluctuations would be completely annihilated. However, better performance is only achieved when a possible reduction in power production, due to the unfamiliar coordination pattern, does not exceed the increase in useful power. Although one might indeed expect lower power production for antiphase rowing we did not find significant differences in *P*_rowers_ between conditions. If any, total power was in fact slightly higher for antiphase rowing. Most importantly, *P*_flywheels_ was significantly higher for antiphase rowing than for in-phase rowing, which indicates that performance was better for antiphase rowing. When generalized to on-water rowing, this would mean that more useful power would be available to overcome drag, resulting in a higher average velocity, hence shorter race time.

### Interpersonal Coordination

While all pairs easily acquired antiphase rowing during the warm-up, one pair’s coordination briefly broke down to in-phase during the maximum effort trial. The rowers of this pair were the least experienced and had also never rowed together before. Initial difficulties in performing the antiphase pattern may of course be overcome by further practicing of antiphase coordination. Indeed, research on bimanual coordination has shown that practicing antiphase coordination resulted in an increase in stability and a reduction in the associated attentional costs [32].

For visually coordinated rhythmic movements it is known that moving in antiphase is usually less well performed than in-phase (e.g., [20]). The higher absolute error (AE*φ*) and standard deviation of relative phase (SD*φ*_PE_) that we observed for antiphase rowing suggests that the same is true for mechanically coupled movements. Although the higher AE*φ* in antiphase rowing can partly be explained by the difference in dwelling around the catch and finish of the stroke (Figure 2B), the higher SD*φ*_PE_ indicates that the decrease in coordinative performance is not solely a consequence of these deviations from harmonicity. While these deviations are inherent to rowing, making the movement more harmonic (e.g. by shifting towards a 1∶ 1 backward-forward movement ratio), would further reduce the net within-cycle movement of the crew’s mass with respect to the boat and hence further increase *e*_v_. However, it is conceivable that such a movement execution negatively affects power output. In this study, the rowers did not adapt towards harmonic antiphase coordination, as shown by ratios of 1∶ 1.2 found in both conditions (see Table 1). The optimal movement execution for antiphase rowing will likely be a trade-off between optimizing power output and minimizing power losses.

### Generalizing from the Lab to the Water

Antiphase rowing is not convenient for two-person boats in which each rower has one oar, because the alternating left-right propulsion would result in large additional yawing motions of the boat. For all other crew disciplines, it is likely that the results obtained on our ergometer setup apply to on-water rowing. Some issues that are not existent in ergometer rowing need to be considered though. For example, the handling of the oars during on-water rowing might result in additional coordination difficulty. Another example is that antiphase rowing requires slightly longer boats [10], because more space is needed between the two groups of rowers opposing their movements. For an eight, one would need about 70 cm extra, which is within the range of commercially available rowing boats. Total shell drag at racing speeds is quite insensitive to such variations in length [33], so slightly increasing boat length would in fact not be disadvantageous. Furthermore, rowing in antiphase pattern might affect the fluid dynamics around the blades. For example, when rowers alternate their strokes, the blades of the four stroke rowers may enter in disturbed water caused by the four bow rowers, which may enlarge the power loss at the blades. Although such issues were beyond the scope of the present study, the current results indicate that it is certainly worthwhile to explore these in the future.

Interestingly, based on analogous reasoning, higher mechanical and energetic efficiency of asynchronous multi-appendage propulsion patterns in water have also been found in locomotion of krill [34]. Along similar lines, studies on biological aquatic locomotion have generally stressed the importance of phase relations of the propulsion movements for hydrodynamic stability and, hence, propulsive efficiency (e.g., [35]–[37]). In sum, this shows that besides maximizing power (or energetic) output, minimizing power (or energy) losses is ubiquitous to biological aquatic locomotion in general.

### Conclusions

In conclusion, this study demonstrated that steady state crew rowing in antiphase coordination on two coupled free-floating rowing ergometers indeed results in better performance, expressed by higher *P*_flywheels_. Antiphase coordination was easily acquired, and despite it being less accurate and more variable than in-phase coordination, this did not negatively affect *P*_rowers_. Thus, a greater fraction of *P*_rowers_ was transferred to the flywheels as a result of the higher *e*_v_, thanks to the smaller velocity fluctuations within each rowing cycle. Practicing antiphase rowing most likely further increases its demonstrated gains. These results argue in favor of the counterintuitive, long-standing out-of-phase rowing myth, thereby encouraging further empirical exploration of (on-water) antiphase crew rowing. On a more general note, this study demonstrates that, although perfectly synchronous coordination may be most stable, it is not necessarily equated with most efficient or optimal performance.
